# Supplementary material for: Are carbon emissions trading and green financial instruments synergistic? -Comprehensive quantitative research based on content analysis
Source: PLoS One. 2024 Mar 7;19(3):e0298601. doi: 10.1371/journal.pone.0298601 (PMC10919601; doi:10.1371/journal.pone.0298601)
Supplement: S1 Data — (ZIP) [file pone.0298601.s001.zip › Data/地级市Z_F工作报告.zip!/─┌├╔╣┼/═¿┴╔╩╨/└┤╫╘╣1⁄2╓┌║┼í╛┬φ┐╦╩2╛▌═°í┐_2013.docx]

各位代表：

现在，我代表市人民政府向大会报告工作，请予审议，并请市政协各位委员和列席会议的同志提出意见。

一、过去五年工作回顾

2008年以来，在自治区党委、政府和市委的正确领导下，市政府团结依靠各族干部群众，深入贯彻落实科学发展观，抢抓战略机遇，应对各种挑战，加快经济发展，保障改善民生，促进社会和谐，圆满完成了三届人大确定的目标任务。

过去的五年，我们坚定不移地转方式、调结构，综合实力和发展后劲不断增强。预计2012年，地区生产总值达到1700亿元，是2007年的近3倍；财政总收入138.1亿元，是2007年的3倍；社会消费品零售总额340亿元，是2007年的2.5倍；完成固定资产投资1320亿元，五年累计完成4120亿元。三次产业比由2007年的20.8：45.7：33.5调整为14：60：26。煤电铝及铝后加工、玉米生物科技、高技术煤化工、农畜产品加工等优势特色产业具备了一定的市场竞争优势和行业影响力。非资源产业占全部工业的比重达到90%。规模以上企业达到631户，销售收入超百亿元的企业2户、工业园区7个。农牧业生产加快向质量效益型转变，新建节水高产高效粮食功能区306万亩，全市粮食产量首次突破200亿斤，实现九连增。肉牛存栏达228万头，畜牧业比重提高5个百分点。农牧业综合机械化水平达到75.2%，农牧业科技贡献率达到50%。服务业发展能力增强，物流、金融、电子商务迅速兴起，在全区率先实现了“万村千乡市场工程”农家店行政村全覆盖，被评为全国流通领域现代物流示范城市和中国金融生态示范城市。城乡一体化进程不断加快，城乡基础设施和公共服务功能不断完善，新增城镇建成区面积60平方公里，城镇化率由35%提高到43.5%。

过去的五年，我们深入推进改革开放，着力打造良好发展环境，保障发展能力不断提高。行政审批体制改革、农村牧区综合改革和社会事业改革稳步推进，建成市旗两级政务服务体系和公共资源交易平台。制定开放合作、培育主导产业、壮大民营经济等政策性文件73份。主动融入东北一体化发展，积极承接发达地区产业转移，累计引资2050亿元。实施了交通、水利、电力等一批基础设施建设项目，提高了发展保障能力。“引乌入通”建设完成，“引绰济辽”项目顺利推进。新建高等级公路751公里，新增公路里程2031公里，新建扩建铁路780公里，新开通航线8条，通航城市达到15个。实施重点生态工程，促进生态系统自然修复，森林覆盖率提高3.3个百分点，达到25.3%，科尔沁沙地呈现从整体良性逆转到局部明显好转的新变化。

过去的五年，我们着力保障民生，加快发展社会事业，群众生活不断改善。城乡居民人均收入实现翻番，分别达到19196元和8731元。整合投入近300亿元，实施富民惠民工程，就学、就医、住房、出行等民生需求得到保障。鼓励和支持创业就业，新增城镇就业8.7万人，被评为国家级创业型城市。基本社会保险和城乡居民最低生活保障标准逐年提高，企业离退休人员养老金人均增加936元，实现城乡居民养老保险制度全覆盖和医疗保险应保尽保。建设保障性住房5.2万套，改造农村牧区危土房8.9万户。解决了123万人的饮水安全问题，55.2万人实现稳定脱贫。基础教育标准化建设得到加强，民族教育质量明显提高，职业教育与市场需求结合更加紧密，在校生扩大近4倍。实施校安工程，改造加固420所学校校舍，建设苏木镇、嘎查村公办幼儿园183所。开展公立医院改革试点，基本药物零差率销售政策有效落实，医院服务功能不断提升。新建卫生院27所、标准化卫生室200所，新农合参合率达到95.8%。科尔沁文化大市建设扎实推进，文化产业得到发展，苏木镇综合文化站和草原书屋实现全覆盖，《安代魂》等50多部文化精品获自治区以上奖励，列入国家“非物质文化遗产”保护目录和传承人数量位居自治区前列。实施事业单位绩效工资改革，工资幅度有较大增长。开展全民健身活动，群众体育和竞技体育水平不断提高。残疾人事业得到新发展，人口和计划生育、人防、外事、侨务、档案、气象、防灾减灾等工作都取得新成绩。

过去的五年，我们积极推进民主法制建设，保持通辽和谐稳定，社会管理不断创新。自觉接受人大依法监督、政协民主监督和社会监督，认真办理人大代表议案、建议和政协委员提案，广泛听取各民主党派、工商联和无党派人士的意见。深入开展精神文明创建活动，公民素质和城乡文明程度不断提高，工会、共青团、妇联和涉老组织作用得到更好发挥。基层民主决策机制逐步建立，城镇社区标准化建设有新进展。普法依法治理取得明显成效，被评为“五五普法全国法制宣传教育先进城市”。创新信访维稳机制，深入排查化解社会矛盾，以群众工作总揽维稳大局，改进群众工作方法，维护群众利益，一大批信访积案得到解决。食品药品安全得到有效保障，安全生产事故明显下降。深入开展严打整治，社会治安形势持续好转，连续十八年获得全区社会治安综合治理暨平安建设先进盟市。认真贯彻党的民族宗教政策，民族团结进步局面得到巩固和加强。军警民共建和双拥工作取得新成绩，连续三次荣获“全国双拥模范城”称号。

刚刚过去的2012年，我们面对经济下行的复杂形势，全力推进“开放转型，创业富民”战略，实施了促发展保增长一系列措施，全市经济社会平稳较快发展。一是优势主导产业实现新发展。煤电铝及铝后加工、玉米生物科技、高技术煤化工、绿色农畜产品加工等产业实现延伸升级。霍林河综合资源循环经济工业园区和通辽自主创新与承接产业转移工业园区集聚效应初步显现。锦联铝板带箔、创源高强高韧铝合金、蒙东能源微电网、奈曼镍铬合金等重大项目取得突破性进展。第三产业规模进一步扩大，蒙东金融城、保税物流中心、内蒙古公共物流信息平台等项目顺利实施。二是现代农牧业迈出新步伐。全面实施“8511521113”工程，新增节水高产高效粮食功能区172万亩，牲畜存栏1773万头只，新增黄牛30万头。退耕还林还草123万亩，压减机电井5052眼。集中力量建设了那仁新农村和白音珠日河新牧区典型。三是对外开放招商引资实现新突破。积极融入东北经济一体化发展，加大对外开放力度，主动承接发达地区产业转移。全市派出专业招商工作组8 个，实施项目610个，引进资金681亿元。四是民生改善取得新成效。重点实施了住房、就业、扶贫等56个民生项目。大学毕业生、退役士兵和困难群体的就业问题得到有效解决。建成自治区首个扶贫信息化管理平台。整合资金21亿元，集中扶持2万贫困户、8万人稳定脱贫。改造农村牧区危土房5.3万户，建设保障性住房2万套，城乡居民住房条件得到改善。五是城市面貌实现新变化。以“迎会创城”为契机，实施主城区“拉大、补欠、提质”工程，建设了辽河公园、胜利河景观带、机场航站楼、民大图书馆广场等138个基础设施和环境改善项目。对民航路、二号村等地段棚户区进行改造，改善了人居环境。沃尔玛城市综合体和碧桂园五星级标准酒店投入使用，孝庄园建成开园。大力推进城市路桥建设，新增改造城市道路130万平方米。实施城市绿化和亮化工程，人均公共绿地面积达到15.5平方米。在主城区开展整治非法客运电动三、四轮车专项行动取得良好效果。通过经营权置换，使民营公交公司11条公交线路的特许经营权收归国有。成功承办了全区第八次精神文明建设经验交流会，通辽人气得到提升，增强了群众的自豪感和幸福感。

各位代表，这五年的成就，是自治区党委、政府和市委正确领导的结果，是全市各族干部群众团结拼搏、共同努力的结果，是各方面关心、支持和帮助的结果。在此，我代表市人民政府，向所有为通辽建设和发展做出贡献的同志们、朋友们，表示衷心的感谢，并致以崇高的敬意！

在看到成绩的同时，我们清醒地认识到发展中还存在一些问题，主要是：综合实力不够强，产业发展不充分，扩总量、转方式、调结构的任务还很重；县域经济发展不平衡，城乡统筹发展任务艰巨；城乡居民收入、基本公共服务和民生保障水平有待进一步提高；社会建设和管理需要不断加强；统筹经济发展与环境保护的力度还需加大；政府自身建设需要进一步加强等等。这些问题，我们必须高度重视，采取有力措施认真加以解决。

过去五年的实践，我们取得了令人瞩目的成绩，积累了宝贵的经验：一是坚持发展第一要务不能动摇。坚定不移地以经济建设为中心，不断做大经济总量。加快转变发展方式，坚持加快发展不松劲、不懈怠，实现经济平稳较快发展。二是坚持培育壮大优势主导产业不能动摇。坚持抓项目、抓园区、抓环境改善，促进产业延伸、产业升级和产业多元。推进工业化、城镇化和农牧业现代化，提升了优势主导产业的接续力和竞争力。三是坚持科技创新和开放带动不能动摇。全力提升科技创新能力，推进全方位、宽领域、深层次的开放合作，为深度调结构、转方式、促发展提供强大动力。四是坚持保障和改善民生不能动摇。在不断创造新的社会财富的同时，坚持富民优先，切实做好住房、就业、扶贫等民生工作，不断增加百姓福祉。五是坚持社会和谐稳定不能动摇。坚持发挥群众在社会建设和管理中的基础作用，改进政府服务方式，坚持依法行政，主动深入基层开展纠纷排查和矛盾化解，畅通诉求表达渠道，切实解决群众最关心、最直接、最现实的利益问题，有效维护社会稳定，为加快发展创造了有利的社会局面。

二、今后五年总体目标和主要任务

今后五年，全市经济社会发展的总体思路是：高举中国特色社会主义伟大旗帜，以邓小平理论、“三个代表”重要思想、科学发展观为指导，深入贯彻党的十八大精神，牢牢把握科学发展主题和转变经济发展方式主线，紧紧围绕“开放转型，创业富民”战略，深化改革开放，强化科技创新，全力保障和改善民生，推动和谐社会建设，着力推进新型工业化、信息化、新型城镇化和农牧业现代化，着力推进县域经济和非公有制经济发展，打造“实力通辽、活力通辽、美丽通辽、幸福通辽”，努力实现全面建成小康社会的宏伟目标。

具体奋斗目标是：

——经济发展更加突出质量和效益。地区生产总值和财政收入实现翻番，科技进步对经济增长的贡献度大幅提升，三次产业协调推进，城镇人口比重每年提高两个百分点以上，城镇失业率控制在4%以下。

——社会和谐程度更加改善。中等收入群体持续扩大，消除绝对贫困现象，城乡居民收入差距和区域发展差距明显缩小。基本社会保障实现城乡全覆盖。

——人民生活更加殷实。城乡居民收入实现翻番。住房保障体系基本形成，人均住房面积达到27平方米以上。人口平均预期寿命有新提高。

——民主法制更加健全。民主形式更加丰富，依法治市进程加快，司法公信力不断增强，

——文化教育更加繁荣。公共文化服务体系基本建成，文化产业增加值占GDP比重、居民文教消费支出比重不断加大。平均受教育年限10年以上。公民道德素质和社会文明程度明显提高。

——资源环境更加节约友好。单位GDP能耗不断下降，主体功能区布局基本形成，科尔沁沙地治理取得重大突破，人居环境明显改善，资源节约型、环境友好型社会建设取得重大进展。

全面建成小康社会，是人民群众之所盼，更是人民政府职责之所在，我们必须力戒空谈、

（一）加快发展转型，着力建设“实力通辽”。始终把经济建设作为首要任务，继续保持高于全区平均水平的增长速度，不断增强综合经济实力，全力打造蒙东经济发展高地，经济总量进入东北地级城市前三位。坚持传统产业新型化、新兴产业规模化、支柱产业多元化，培育壮大“十大支柱产业”，增强县域经济发展实力；按照“坚持城乡统筹一体化发展，加快农牧业结构调整和经济转型，加快农牧民增收致富，加快农村牧区全面达小康”的总体要求，提升农牧业现代化发展水平，打造全国领先的规模化、集约化、标准化现代农牧业示范区，实现粮食功能区节水设施全覆盖，建成全国肉牛产业第一市和国家绿色食品安全示范市；围绕“两个基地、一个中心”，加快现代服务业发展，建设优质安全高端绿色农副产品生产基地、旅游观光休闲度假基地和区域物流中心，构建立足蒙东、面向东北、承东接西、贯通南北的大物流格局。把增强文化软实力作为重要发展战略，打造特色文化产业园区，培育文化龙头企业，形成具有通辽特色的文化优势，文化产业主要发展指标跃居自治区前列，努力建设一个经济实力足够强大、人民生活足够充裕、文化生活足够丰富的实力通辽。

（二）增强创新驱动，着力建设“活力通辽”。以打造蒙东科技高地为目标，加强科技合作交流，加快科技与产业、科技与文化、科技与金融相结合，探索引进和培养人才的新机制，不断增强自主创新能力。全面落实重点领域和关键环节的改革举措，大力发展非公有制经济，为非公经济发展创造良好环境，充分激活民间资本和各类发展要素，实现市场主体总量翻番。全面融入东北经济一体化发展，加强面向全国和周边国家的务实合作，加快人流、物流、信息流和资金流的深度融入互动，提高对外开放的综合水平，提升经济发展的活力和

（三）推动可持续发展，着力建设“美丽通辽”。按照建设国家生物多样性示范城市的要求，充分发挥3810万亩林业用地的生态和经济功能作用；科学保护和利用好5129万亩草牧场；加大1106万亩自然保护区的管护力度；规划治理5000万亩科尔沁沙地，推进“四大生态功能区”建设，使三分之二的国土得到自然修复，保护生物多样性。加强水源地保护和用水总量管理，大幅降低能源、土地等消耗强度，走绿色发展、循环发展、低碳发展之路。改善和优化城乡人居环境，推进城乡基础设施和公共服务一体化发展，突出产业支撑、功能配套、服务完善。建设各具特色的生态型城镇，每年建设100个新农村新牧区示范村，同步建设绿化示范村。拓展城市发展空间，完善城市功能，打造科尔沁百万亩森林城市，建设东北第一生态城，创建国家森林城市和国家园林城市。

（四）促进创业富民，着力建设“幸福通辽”。关注人民的生活质量和幸福指数，拓宽增收渠道，实现群众收入与经济发展同步增长；实施积极的就业政策，促进大学毕业生和困难群体就业，使城乡就业更加充分；提高保障标准，建成覆盖城乡居民的社会保障体系和城乡困难群体救助体系；深入推进连片重点扶贫开发，贫困农牧民年均增收20%以上，36.6万贫困人口稳定脱贫；大力改善住房条件，全市保障性住房覆盖面达到25%以上，全面完成农村牧区危房改造任务；优化教育资源配置，提高农村牧区义务教育水平；加快建设覆盖城乡的公共卫生服务体系，人人享有基本医疗服务，食品药品安全得到有力保障；完善农村牧区交通设施和城市公交体系，方便群众出行；创新社会管理体制机制，维护社会和谐稳定，努力建设人民群众有归属感、自豪感的幸福通辽。

今年经济社会发展的主要预期目标是：地区生产总值增长16%，地方财政收入增长14%，全社会固定资产投资增长22%，社会消费品零售总额增长20%，城乡居民人均收入分别增长14%和18%，城镇登记失业率控制在 4%以内，人口自然增长率控制在7‰以内，完成年度节能减排目标任务。

（一）加快工业提质增效，实现转型发展新提升

继续加大投资力度。发挥投资和项目建设对推动我市经济持续健康发展的基础性作用，抓住结构调整的关键环节和领域，从投资源头上抓好结构调整，重点向基础设施建设、产业建设、城乡建设、生态建设和民生建设等领域倾斜，通过政府引导性投资、扩大市场融资、引导民间投资、积极招商引资等方式，促进投资主体多元化，多渠道筹集资金，力争今年全社会固定资产投资完成1600亿元。确定今年为“重大项目突破年”，切实增强全市抓项目、服务项目的自觉性、主动性和坚定性，使各类项目尽快转化为现实生产力。

培育壮大主导产业。进一步发挥资源、能源等比较优势，做好科技创新和循环发展文章，推动传统产业新型化、新兴产业规模化、支柱产业多元化，培育煤电铝及铝精深加工、玉米生物科技、绿色农畜产品加工、高技术煤化工、新型建材、装备制造、镍铬合金、现代蒙药、新能源和新材料等“十大支柱产业”。实施重点工业项目300个以上，工业固定资产投资达到750亿元以上，全部工业增加值突破1000亿元。加快铝合金及铝后加工、新型材料项目建设，推进煤电用一体化循环发展进程，打造全国铝加工基地；推进氨基酸、聚乳酸、维生素及其下游系列产品开发，加快玉米生物科技产业延伸提质，打造世界级氨基酸生产基地；推进煤制乙二醇、苯胺、煤制气等项目建设，加快谋划高技术煤化工产业延伸多元项目；发挥绿色农畜产品基地和特色优势，打造科尔沁肉牛、红干椒、荞麦系列产品生产加工基地；大力发展风电设备制造、汽车模具及零部件、矿山机械、农业机械等装备制造产业，提高成套加工能力和基础配套水平；推进镍铬合金及配套项目建设，加快培育煤电冶建循环发展产业链条；构建现代蒙药、生物制药的自主创新、生产制造、流通服务体系，推进蒙药工业园区建设，构建现代蒙药科研生产基地；加快推进风力、光伏、风光互补和生物质发电项目，建设千万千瓦级风力发电基地和百万千瓦级光伏发电基地；做强煤电硅和光伏材料制造产

以园区建设为重点提高县域经济发展水平。坚持集约集群化发展，完善园区功能，搭建县域经济发展平台。促进产业向园区集中，霍林河综合资源循环经济工业园区，重点培育煤电铝及铝后加工、高技术煤化工及配套建设物流等产业；通辽自主创新与承接产业转移工业园区，重点培育玉米生物科技、绿色农畜产品加工、有色金属加工、装备制造等产业，两大增长极力争产值均达到1000亿元。推动旗县重点工业园区提档升级，提高承载能力，促进产业培育、园区建设、城镇建设协调发展，强化对县域经济的龙头带动作用。重点培育优势特色园区，每个旗县市区都要在现有园区和产业基础上，更加突出自身发展特色，使园区成为展示县域经济发展的平台和亮点。全市15个旗县工业园区入驻企业突破1000户，旗县8个重点园区产值均超百亿元。

积极促进企业发展成长。支持具备条件的企业申报自治区和国家级企业技术中心，积极创建自主知名品牌，建设自治区级小企业创业基地和公共服务示范平台，加快培育优势产业集群和大企业集团。大力实施开发性融资，加强与驻市各金融机构的协调，降低信贷门槛，增加贷款规模，密切银企对接，强化金融服务实体经济功能。突出抓好平台融资和直接融资。重点做好市城投公司二期20亿元地方债券发行工作，加快推进蒙药公司等符合条件的企业上市，逐步提高直接融资在全市融资中的比重。全年确保社会融资总量余额突破1000亿元。

（二）提升现代农牧业发展水平，促进农牧民稳定增收

按照“一个坚持，三个加快”的总体布局要求，坚定不移地推进“8511521113”工程，不断提高农牧业综合生产能力。

发展节水高产高效农业。整合农牧业项目资金，强化科技支撑，突出重点区域，集中推进粮食功能区建设，新建节水高产高效示范区107万亩，粮食功能区建成总面积达到413万亩。新增设施农业面积12万亩，总面积达到65万亩。启动11个安全食品产业示范带建设，推动红干椒、荞麦、沙地西瓜等特色产业提质升级。实施节水示范和高标准基本农田建设工程，新建4个旗县级农田灌溉和水资源信息化管理示范区，压减机电井8300眼，节水3.1亿立方米。

提升建设型畜牧业发展水平。强化肉牛、肉羊、生猪等养殖基地建设，扶持养殖大户，鼓励龙头企业自建养殖基地，牲畜存栏2050万头只。强化饲草料基地建设，新增紫花苜蓿种植面积50万亩，全株青贮玉米饲草料基地达到325万亩，秸秆转化利用率达到70%。强化肉牛加工龙头企业、养殖大户、农牧民分户饲养三个层面工作，新增肉牛存栏32万头，农牧民人均肉牛养殖纯收入达到1300元，巩固提升科尔沁肉牛产业品牌优势。

加快推进新农村新牧区建设。坚持统一规划、突出特色，完成100个新农村新牧区暨民族团结进步示范嘎查村建设，推动基础设施、产业项目、公共投入、公共服务优先向示范村倾斜，实现水、电、路、房等7个百分百，科技、教育、文化、卫生、商贸、金融、社保等20项全覆盖。积极探索农牧民进城新体制，完善土地流转、户籍管理、就业安置、住房保障等相关政策，为农牧业转移人口市民化创造条件。

完善农牧业生产经营管理体制。探索农牧业规模化、标准化、信息化、市场化新机制，加强新型专业合作社建设，突出抓好146个农机种植专业合作社，扶持100个养殖专业合作社发展。开展嘎查村集体经济组织公司化改造试点，推动产、商、科、融一体化经营。建立完善农牧业灾害预警预报制度，加强农牧业病虫害防治、重大动物疫病防控、科技推广体系建设，提高农牧业防灾减灾能力。

加大区域协调发展和扶贫开发力度。着眼于区域发展带动脱贫致富，解决城乡差距、南北差距问题，重点加大对南部四个国贫旗的扶持力度，确保贫困人口减少7万人、人均纯收入增长20%以上。集中扶持科左中旗5000户、2万人稳定脱贫。

（三）壮大现代服务业，拓宽结构优化新路径

做大做强商贸物流业。按照打造东北区域物流中心目标，进一步优化商贸物流业空间布局，提高对财政、就业的贡献度。做大煤炭、粮食、建材、工业原材料和产成品等大宗物流，培育冷链物流、电子商务等新型业态，引进第三方物流企业，拓宽物流服务领域。推进以主城区“四园一港”为重点的11个物流园区和60个重点项目建设。保税物流中心保税仓库投入运营，扩大通辽内陆港集装箱发运量，提升内蒙古物流公共信息平台、粮食交易中心电子交易平台服务功能。加强城乡流通体系建设，改善消费环境，提升消费水平。推进现代农畜产品市场体系建设，开展“农超对接”和“便民服务进社区、进家庭”活动，抓好商务部家政龙头企业试点城市工作。全市货运总量增长20%，物流业增加值实现300亿元。

做大做强文化旅游业。深度挖掘文化旅游资源，规划建设“一轴三区”500公里文化旅游带。抓好科尔沁马城、后旗乌丹五角枫旅游园等特色文化产业园建设，开工建设哈民史前聚落遗址、南宝力皋吐史前古墓群遗址博物馆，复建僧格林沁亲王府，开工建设塔敏查干沙漠旅游项目。开展与中青旅控股股份有限公司的务实合作，加快重点旅游景区提档升级，培育特色旅游精品线路，扩大旅游交流合作，融入全国旅游大市场。力争孝庄园通过4A级景区验收，大青沟景区和库伦三大寺通过5A级景区验收。办好中国蒙古族服装服饰艺术节暨蒙古族服装服饰大赛，提高我市知名度。全年接待国内外游客500万人次，旅游总收入130亿元。

鼓励发展新兴服务业。加强政银企合作，引导金融机构做大总量、优化结构、创新金融产品，多渠道为重点项目和中小企业提供金融服务。实施农村牧区金融改革试点工作，完成农村商业银行组建工作，扩大农业保险覆盖面。推进光大银行分行、民生银行村镇银行开业运营，增加小额贷款公司、融资性担保公司批筹和营业数量。加快蒙东国际金融城建设，推进蒙药集团上市融资。整合高职院校、科研机构和企业研发中心资源，建设科技与创新产业孵化器，促进现代服务业和先进制造业融合发展。扶持发展物业管理、家政服务、医疗保健、社区养老等社区服务业。推进发展非基本医疗服务，鼓励有条件的公立医院和机构开展医疗护理、健康检查、卫生保健、康复护理等健康服务。加快发展会计事务、管理咨询、人才培训等中介服务业。积极引进高端会展品牌，培育发展会展经济。

（四）推动城乡一体化发展，构筑新型城镇化格局

加快推进百万人口区域中心城市建设。完成城市总体规划修编，开展城市综合交通、城市生态绿地系统、城市供水等专项规划编制，完成城东、民航路、铁南片区控制性详规。推进“拉大、补欠、提质”工程建设，重点实施科技馆、城市展览馆等公共建筑、新世纪大桥、孝庄河景观带、百万亩城郊生态林、万达广场、科尔沁马城等重大城建工程。改造红光大街、民主路等15条街路和60条小街小巷，完成一批供热、电力、给排水、污水污泥处理和老旧住宅楼改造项目，大规模推进棚户区改造。实施绿化景观工程，种植大树6万株，主城区绿化覆盖率达到45%。深入开展“公交规范运营和文明服务年”、“城市建设管理年”和创建“国家级卫生城”活动，建设宜居、宜业、宜游、宜购的区域性中心城市。

加快推进城关镇和重点镇建设。完成城关镇总规修编和重点镇总体规划编制工作。加快县城扩容提质步伐，突出抓好旧城改造和新区建设。实施新型城乡统筹示范工程，加大城中村、城边村改造力度，稳步推进城乡一体化进程。

加快推进覆盖城乡的基础设施建设。加快实施续建铁路项目，推进京通线电气化扩能改造、通辽至京沈客专联络线建设。启动通辽至鲁北高速公路建设，确保好通高速公路、通辽至凤凰岭、大林至保康、甘旗卡至库伦一级路建成通车，开工建设霍林河至阿力得尔、舍伯吐至白音胡硕一级公路、乌拉盖至霍林河二级公路。实施好全国农村公路建设示范试点，建设通村水泥路1500公里。加快霍林河支线机场建设，推动通辽机场升级为蒙东支线枢纽机场，旅客吞吐量达到50万人次以上。推进电力、天然气等能源输送通道建设。

（五）推进科技创新和改革开放，增添加快发展新活力

推动科技创新体系建设。探索建立科技成果转化服务平台，深化与清华大学、内蒙古师范大学、内蒙古民族大学、国机集团的产学研合作，完善科技要素交易市场，提高科技成果转化率。实施科技创新计划和科技“双十”工程，落实重大科技专项，在铝新材料、煤化工新产品、现代蒙药、肉牛产业、节水农业、种子工程、治沙、秸秆转化利用等方面进行重点攻关，完善玉米、肉牛、红干椒、蒙药等技术标准和规范。鼓励企业设立研发中心，推进畜牧研究院、院士工作站等工程建设，促进科技与产业紧密结合。

大力发展非公有制经济。毫不动摇地鼓励、支持、引导非公有制经济发展，按照“非禁即入、平等待遇”的原则，进一步拓宽民营经济投资领域和范围。抓好《市政府关于促进民营经济又好又快发展意见》的落实，加大对非公经济的服务力度，激发各类市场主体发展活力，促进民营经济快速发展壮大。今年市场主体数量增长25%以上。

提高对外开放水平。推动与东北地区的深度融合发展，抓好与东北地区合作协议和项目的落实，巩固扩大与发达地区的对接合作，在引进母体企业、承接整产业链转移上实现新突破。加强与自治区工商联的对接联络，吸引更多的投资者来我市兴业发展。推进与周边国家和地区的经贸合作，努力形成多层次、多领域、多元化的对外开放新格局。主动承接长三角、珠三角、京津冀、港澳台等发达地区产业转移，实施产业链配套招商。坚持一手抓项目引进，一手抓落地履约，确保项目引得进来、落得下去，形成真正的实物投资量。进一步完善招商引资优惠政策，提高市场开放度，建设蒙东地区对外开放的桥头堡。

（六）加强生态环境建设与保护，拓展可持续发展空间

抓好《通辽市2013年生态文明建设行动计划》的落实，实施生态移民和收缩转移战略，不断增强可持续发展能力。加强自然保护区管理，推动罕山、双合尔山自然保护区分别晋升为国家和自治区级自然保护区。完成林业生态建设206万亩，实施退耕还林还草154万亩，综合治理科尔沁沙地63万亩，建设主要通道绿化带和100个绿化示范村。突出抓好葡萄和饲草等沙地产业发展，实现增绿与增收双赢。严格水资源管理，提高水资源保障能力，增强枢纽调节功能，扩建孟家段水库和莫力庙水库进水渠，新建及除险加固一批中小型水库，全力推进“引绰济辽”等重大跨流域调水工程。

严把新上项目准入关，从源头上控制能耗不合理增长。狠抓重点领域、行业、企业节能减排，集中实施工业重点节能技改和资源综合利用项目，推进合同能源管理试点工作，促进中水回用、风电供热等节能减排新技术推广应用。加强城乡环境综合治理，重点解决空气、土壤、水源污染等环境问题。倡导循环低碳生产生活方式，形成节能环保的良好氛围。

（七）提升公共服务水平，推进民生得到新改善

保障和改善民生。整合各级民生项目资金，着重做好民生30件实事，切实增强惠民实效。城乡居民低保补助水平每月分别提高40元和25元。提高企业退休人员基本养老金和城镇基本医疗保险待遇水平，推进城镇职工和居民医保门诊统筹，开展城镇居民大病保险试点工作。实现新农合市级统筹，筹资标准提高到每人每年340元，最高支付限额提高到20万元。新建保障性住房1.24万套，改造农村牧区危房2万户以上。

办好人民满意的教育。下力量解决择校热、大班额、入园难等突出问题，保障“留守儿童”和进城务工人员随迁子女平等接受教育。新增农村牧区幼儿园30所，完成学前教育三年行动计划。完善现代民族教育体系，实施市蒙古族中学迁址和市民族幼儿园建设工程。支持职业教育发展，加快主城区职业学校整合，改善旗县职业学校办学条件。抓好教师队伍建设，加强教育督导考评，着力提高教育教学质量。

做好就业增收工作。发挥企业吸纳就业主渠道作用，完善企业职工工资集体协商制度，大力开发社区保障、社区养老等公益性岗位，创造更多的就业机会。深入实施创业带动就业工程，建立创业孵化基地，强化创业培训，突出做好高校毕业生、农村牧区转移劳动力、城镇困难人员、退役军人就业工作。新增城镇就业1.6万人。鼓励增加合法收入，提高中低收入者的收入水平，规范提高财政供养人员津贴标准。

发展卫生事业。建设20所边远贫困地区卫生院和500所标准化嘎查村卫生室，旗县医院全部达到二甲以上等级标准，支持市级医院与知名医院建立合作关系，实现基本医疗卫生服务全覆盖。加强通辽市医院重点学科建设，积极引进先进医疗设备和人才。加强自主研发、药材基地建设和经典传承，设立蒙医药发展基金，扶持蒙中医药事业发展，争创全国蒙中医先进市。启动新城区健康体检中心、肿瘤医院和民大附属蒙医医院建设。强化优生促进工作，提高出生人口素质，创建国家卫生城市。

推进文化等社会事业发展。加强非物质文化遗产传承和保护工作，着力打造草原文化品牌，推出一批具有科尔沁民族特色的文艺精品。支持传承民族传统工艺，研发文化特色鲜明的创新设计和产品。以市文化产业公司为龙头，加快文化旅游资源整合和产业化步伐。加快推进重点文化惠民工程，完成直播卫星“户户通”工程和通辽自办蒙汉语广播节目农村牧区覆盖工程，建设嘎查村级文化广场200个、文化室600个。广泛开展全民健身活动，提高竞技体育运动水平。加强新闻出版、人防、外事、侨务、档案、史志、气象、防灾减灾等工作，维护老年人、妇女、儿童和残疾人合法权益。

（八）加强和创新社会管理，推进和谐通辽建设

提高社会管理水平。抓好社会管理创新综合试点工作，推行“532”基层民主决策监督管理机制、“社区网格化管理”等模式，强化基层自治和服务功能，规范新社会组织，创新流动人口和特殊人群管理服务。强化网络管理，推进网络依法规范有序运行。加强法律援助和人民调解工作，建立重大决策社会稳定风险评估机制，切实把矛盾纠纷防范在事前、解决在基层。加强国防教育、国防动员、“双拥”和军队转业干部安置工作。

营造安全稳定的社会环境。推进立体化社会治安防控体系和数字化警务平台建设，提高公安机关精确打击犯罪能力，净化社会治安环境。严格落实安全生产责任制，加强对高危行业和重点领域的隐患排查治理，坚决遏制重特大安全事故发生。健全完善防灾减灾和应急避险管理体系，有效防范和处置突发性和群体性事件。全面实施食品药品安全放心工程，完善突发卫生公共事件应急和重大疾病防控机制，切实保障人民群众生命健康。

加强精神文明和民主法制建设。深入开展社会主义核心价值体系教育，努力提高公民道德素质和全社会文明程度。全面贯彻党的民族政策，推进民族聚居地区和民族团结进步示范村建设，促进各民族和睦相处、和谐发展。主动接受人大法律监督、政协民主监督和社会监督，认真办理人大代表的议案、建议和政协委员提案。深入开展“六五”普法工作，增强依法行政和公正司法能力。

四、加强政府自身建设

各位代表，站在新的历史起点上，新一届政府肩负着全面建成小康社会的光荣使命，任务艰巨，责任重大。我们要深入贯彻落实党的十八大精神，遵照中央政治局提出的八项规定要求，牢牢记住“空谈误国、实干兴邦”，切实转变工作作风，加强政府自身建设，以崭新的精神风貌更好地服务于经济社会发展。

加强服务型政府建设，转变政府职能。坚持以人为本，执政为民，加快政府职能由管理型向服务型转变，把群众的冷暖放在心上，把企业的发展放在心上，全力为民生需求和企业需求服务好。以提高服务质量和效率为重点，扩大政务公开范围，提升公开时效性，把政务服务中心和公共资源交易中心建设成依法、高效、阳光、温馨的政务服务平台。

加强创新型政府建设，转变工作作风。大兴求真务实之风，做到讲实话、干实事、勇担当、负责任。完善领导干部调查研究和联系基层制度，面对面解决实际问题。对研究决定的事抓住不放，强化落实，务求实效。进一步改进会风文风，严格控制政府会议的数量和规模。

加强廉洁型政府建设，树立政府公信力。严格执行“三重一大”集体决策制度，加强对行政权利的监督制约。实党风廉政建设责任制，强化领导干部公仆意识，把更多的时间和精力用在研究工作、破解难题、维护稳定、推动发展上。着力解决好土地征用、房屋拆迁、环境保护、工程招投标等领域中的突出问题，加大行政监察和审计监督力度，严厉查处失职渎职行为。厉行勤俭节约，反对铺张浪费，确保“三公”支出零增长，严格控制各类节庆活动，真正做到为人民服务，对人民负责，受人民监督，让人民满意。

各位代表，宏伟蓝图鼓舞着我们，全市人民信任期待着我们。让我们在自治区党委、政府和市委的正确领导下，振奋精神，扎实工作，全力推进“开放转型、创业富民”战略，为建设实力通辽、活力通辽、美丽通辽、幸福通辽，为全面建成小康社会而努力奋斗！
